# State Legislation—New York

**Published:** 1895-02

**Authors:** 


### STATE LEGISLATION.

### NEW YORK.

New York veterinarians are desirous of amending the present laws relating to and governing the practice of veterinary science in that State.

Under qualifications for practice the first requirement after July 1, 1896, shall necessitate a license by the Board of Regents of the University of the State of New York.

Secondly, that there shall be established a State board of veterinary medical examiners, of five members, to hold office for three years. These to be appointed from nominations made by the New York State Veterinary Medical Society, or, on their failure, the Regents may appoint from members in good standing in the veterinary profession without restriction. Each member must be a graduate of a registered veterinary medical school and have practised for at least five years. They shall be removable for cause.

A third section provides for a certificate of appointment, oath of office, and powers, subject to the approval of the Regents.

A fourth section provides for expenses, the board of examiners only receiving compensation from any funds remaining after all expenses are paid.

The fifth section provides for officers of the board and the necessary meetings, a majority to constitute a quorum, and the assignment to committees of examining papers and candidates.

A sixth section as to admission of examination : The Regents shall admit to examination any candidate who pays a fee of $25 and submits satisfactory evidence, verified by oath if required, that he: (1) is more than twenty-one years of age; (2) is of good moral character; (3) has the general education required in all cases after July 1, 1896, preliminary to receiving a degree in veterinary medicine; (4) has studied veterinary medicine not less than three full years, including three satisfactory courses in three different academic years in a veterinary medical school registered as maintaining at the time a satisfactory standard; (5) has received a degree as veterinarian from some registered veterinary medical school. The degree in veterinary medicine shall not be conferred in this State before the candidate has filed with the institution conferring it the certificate of the Regents that three years before the date of the degree, or before or during his first year of veterinary medical study in this State, he has either graduated from a registered college or satisfactorily completed not less than a three-years' academic course in a registered academy or high school; or has a preliminary education considered and accepted by the Regents as fully equivalent; or has passed Regents' examination in arithmetic, elementary English, geography, spelling, United States history, English composition, and physics. Students who had matriculated in a veterinary medical school before October 5, 1893, shall be exempted from this preliminary education requirement, provided the degree be conferred before July 1, 1896. The Regents may, in their discretion, accept as the equivalent for any part of the third and fourth requirement evidence of five or more years' reputable practice in veterinary medicine, provided that such substitution be specified in the license.

A seventh section provides for at least four stated examinations yearly, and in at least four convenient places, and that all examinations shall be exclusively in writing and in English. Examination to be conducted by a Regent examiner; the candidate's papers to be submitted to the board of State examiners; candidates failing are accorded a second examination after six months without fee, this time to be spent in study.

An eighth section, as follows: On receiving from the State board an official report that an applicant has successfully passed the examination and is recommended for a license, the Regents shall issue to him, if in their judgment he is duly qualified therefor, a license to practise veterinary medicine. Every license shall be issued by the University under seal, and shall be signed by each acting veterinary medical examiner of the board, and by the officer of the university who approved the credential which admitted the candidate to examination, and shall state that the licensee has given satisfactory evidence of fitness as to age, character, preliminary and veterinary medical education and all other matters required by law, and that after full examination he has been found properly qualified to practise. Applicants examined and licensed to practise before July 1, 1896, by other State examining boards registered by the Regents as maintaining standards not lower than those provided by this article, and applicants who matriculate in New York State veterinary medical schools before July 1, 1896, and who receive the veterinary degree from a registered veterinary medical school before July 1, 1896, may, without further examination, on payment of $10 to the Regents, and on submitting such evidence as they may require, receive from them an indorsement of their license or diplomas, conferring all rights and privileges of a Regents' license issued after examination. If any person whose registration is not legal on account of some error, misunderstanding, or unintentional omission, shall submit satisfactory proof that he had all requirements prescribed by law at the time of his imperfect registration, and was entitled to be legally registered, he may, on unanimous recommendation of the State board of veterinary medical examiners, receive from the Regents under seal a certificate of the facts, which may be registered by any county clerk, and shall make valid the imperfect registration. Before any license is issued it shall be numbered and recorded in a book kept in the Regents' office, and its numbers shall be noted on the license. This record shall be open to public inspection, and in all legal proceedings shall have the same weight as evidence that is given to a conveyance of land.

A ninth section provides for the proper registration of such licensed veterinarians in the various counties of the State.

A tenth section provides for registration in another county or more than one county.

An eleventh section—certificate and presumptive evidence—unauthorized registration and license prohibited. Every unrevoked certificate and indorsement of registry, made as provided in this article, shall be presumptive evidence in all courts and places that the person named therein is legally registered. Hereafter no person shall register any authority to practise veterinary medicine unless it has been issued or indorsed as a license by the Regents. No diploma or license conferred on a person not actually in attendance on the lectures, instructions, and examinations of the school conferring the same, or not possessed at the time of its conferment of the requirement then demanded of veterinary medical students in this State as a condition of their being licensed so to practise, and no registration not in occordance with this article, shall be lawful authority to practise veterinary medicine, nor shall the degree of doctor of veterinary medicine be conferred *causa honoris* or *ad eundum*, nor if previously conferred shall it be a qualification for such practice.

A twelfth section—construction of this article—shall not be construed to affect commissioned veterinary medical offices serving in the United States Army or in the United States Bureau of Animal Industry while so commissioned; or any person for giving gratuitous services in cases of emergency; or any lawfully qualified veterinarian in other States or counties meeting legally registered veterinarians in this State in consultation , or any veterinarian residing on a border of a neighboring State and duly authorized under the laws thereof to practise veterinary medicine therein, whose practice extends into this State, and who does not open an office or appoint a place to meet patients or receive calls within this State; or any veterinarian duly registered in one county called to attend isolated cases in another county, but not residing or habitually practising therein. This article shall be construed to repeal all acts or parts of acts authorizing conferment of any degree in veterinary medicine, *causa honoris* or *ad eundum*, or otherwise, than on students duly graduated after satisfactory completion of a preliminary and veterinary medical course not less than that required by this article as a condition of license.

The thirteenth section provides for penalties for violation of any sections or section of this act.

